# Mitochondrial DNA Variation and Cognitive Function in Mid‐life

**DOI:** 10.1002/alz70855_099985

**Published:** 2025-12-23

**Authors:** Nien Yeh Daphne Yu, Shea J Andrews, Lifang Hou, Kristine Yaffe

**Affiliations:** ^1^ Department of Epidemiology and Biostatistics, University of California, San Francisco, San Francisco, CA, USA; ^2^ Department of Psychiatry and Behavioral Sciences, University of California ‐ San Francisco, San Francisco, CA, USA; ^3^ Northwestern University Feinberg School of Medicine, Chicago, IL, USA; ^4^ Department of Psychiatry, Neurology, and Epidemiology and Biostatistics University of California San Francisco School of Medicine, San Francisco, CA, USA

## Abstract

**Background:**

Mitochondrial dysfunction has been implicated in various age‐related health issues and neurodegenerative disorders, but the role of mtDNA mutations is underexplored, especially in younger and more diverse populations. One key aspect of mtDNA is heteroplasmy, the presence of more than one allele in mtDNA, which may contribute to mitochondrial dysfunction. Additionally, mitochondrial haplogroups are maternally inherited groups of mtDNA variations that reflect ancestral lineages and may influence disease risk. Here, we evaluated the effects of mitochondrial heteroplasmy (mtHz) burden and mitochondrial haplogroup (mtHg) on cognition at mid‐life.

**Method:**

We studied 2308 participants from the Coronary Artery Risk Development in Young Adults study (mean age 45.25 ± 3.55 years, 58.2% female, 45.5% Black, and 55.5% White at baseline). Heteroplasmy burden 5 years from baseline was measured by the sum of Mitochondrial Local Constraint Score, which takes each variant's observed‐to‐expected ratio adjusted by its homoplasmy count in population to calculate the constraint effect of the variant. Haplogroups were determined with reference to PhyloTree Build 17. We conducted multivariate linear regression to investigate associations of mtHg and mtHz to cognitive outcomes assessed 10 years from baseline, adjusting for baseline age, sex, race, and years of education. Race‐stratified analysis was conducted to determine mtHg effects within race, with common haplogroups H and L3 as reference for White and Black participants respectively.

**Result:**

There was no association between heteroplasmy burden and cognitive function. Haplogroups in macro‐haplogroup L was significantly associated with worse cognitive outcome across all participants, particularly in processing speed and global cognition. mtHg N was associated with improved processing speed among White participants, and with better executive function, verbal memory, and global cognition among Black participants. mtHg K was associated with worse executive function among White participants. However, none of the race‐specific associations were significant after adjusting for multiple comparisons.

**Conclusion:**

Our findings suggest potential associations between mtHg and cognitive performance, pointing to mtDNA variation as a possible factor of mid‐life cognitive health. Race‐stratified results underscore the need to account for different haplogroup distributions within racial groups and warrants further investigation to clarify the underlying genetic, environmental, and social contributors.

